# An Integrated Analysis of Anatomical and Sugar Contents Identifies How Night Temperatures Regulate the Healing Process of Oriental Melon Grafted onto Pumpkin

**DOI:** 10.3390/plants13111506

**Published:** 2024-05-30

**Authors:** Huan Liang, Jiangfeng Liu, Xianfeng Shi, Mihong Ge, Juhong Zhu, Dehuan Wang, Mobing Zhou

**Affiliations:** Wuhan Academy of Agricultural Sciences, Wuhan 430070, China; lianghuanconf@126.com (H.L.); ljf980223@163.com (J.L.); shixf124@163.com (X.S.); gmh917@126.com (M.G.); hongye408@163.com (J.Z.); wdhuan1987@163.com (D.W.)

**Keywords:** temperature, melon, healing process, anatomical, sugar

## Abstract

Graft healing is a complex process affected by environmental factors, with temperature being one of the most important influencing factors. Here, oriental melon grafted onto pumpkin was used to study changes in graft union formation and sugar contents at the graft interface under night temperatures of 18 °C and 28 °C. Histological analysis suggested that callus formation occurred 3 days after grafting with a night temperature of 28 °C, which was one day earlier than with a night temperature of 18 °C. Vascular reconnection with a night temperature of 28 °C was established 2 days earlier than with a night temperature of 18 °C. Additionally, nine sugars were significantly enriched in the graft union, with the contents of sucrose, trehalose, raffinose, D–glucose, D–fructose, D–galactose, and inositol initially increasing but then decreasing. Furthermore, we also found that exogenous glucose and fructose application promotes vascular reconnection. However, exogenous sucrose application did not promote vascular reconnection. Taken together, our results reveal that elevated temperatures improve the process of graft union formation through increasing the contents of sugars. This study provides information to develop strategies for improving grafting efficiency under low temperatures.

## 1. Introduction

In vegetable crops, grafting has been commonly used for counteracting adverse cultivation situations, including temperature changes, organic pollutants, heavy metals, high salinity, drought, insect pests, and soil-borne pathogens [1,2]. Grafting is considered to be a cost-effective and environmentally friendly operation. In addition, this technique improves fruit yield and productivity by enhancing water use efficiency and nutrient uptake [3]. Worldwide, a high percentage of grafted *Cucurbitaceae* and *Solanaceae* vegetables has been widely applied in agricultural practice [4]. However, environmental stress at the graft union formation stage leads to a low survival rate.

It is widely recognized that the successful healing of rootstocks and scions is a crucial prerequisite for the cultivation of grafted seedlings. Successful grafting, involving the initial adhesion of scion and rootstock, callus tissue formation, and vascular reconnection, depends on internal factors, including hormones, sugars, and the developmental stage, but also on external factors, such as temperature, humidity, light, and culture methods [5,6,7]. Optimizing environmental factors during the healing and acclimatization stages is essential to produce high-quality grafted seedlings. One notable factor is temperature, which influences the growth rate of the plant, the rate of wound healing, and regeneration. In Walnut, the graft success rate increased from 6% to 73% with localized heating [8]. In Arabidopsis, raising the healing temperature from 22 °C to 27 °C speed up grafting and vascular reconnection by approximately 25% [9]. In tomato, the tensile strength of the graft union was greatly improved when the stored temperature of the graft union ranged from 23 °C to 27 °C [10].

Melon (*Cucumis melo* L.) is an important horticultural crop that belongs to the *Cucurbitaceae* family. In China, melon plants are mainly grafted during in early spring, when plants often suffer from low temperatures. The night healing temperature decreases to 12 °C, which results in a wider gully between the rootstock and scion cut surface and delays the differentiation of vascular tissue, leading to unsuccessful graft union formation [11]. The optimal healing temperature ranges from 22 °C to 28 °C [12,13]. In agricultural practice, heating is necessary to ensure the high survival rate of the grafted seedlings. However, heating is interlinked with energy consumption and has economic impacts. So, understanding how temperature regulates the biological processes during graft union formation under low-temperature conditions is crucial to achieve a sustainable agricultural system.

Sugars are active and essential during graft union formation [14]. Sugars, in addition to their fundamental roles as carbon and energy sources, also act as signaling molecules to regulate gene expression. Almansa [15] found that the graft healing ability of macadamia nuts was influenced by the carbohydrate content of the scion. Marsch-Martínez [16] found that adding 0.5% sugar to the grafting medium resulted in faster recovery after grafting and a higher graft success rate compared to plants with 0% sugar. However, the function of carbohydrates in graft union formation under low temperatures has not yet been studied.

To reduce the impact of external environmental factors and improve the healing efficiency of melon grafted plug seedlings, we performed an anatomical analysis and integrated metabolic analysis to observe the cellular and metabolite changes that occur during graft union formation. Furthermore, the application of exogenous glucose, fructose, or sucrose demonstrated that glucose and fructose play important roles in melon graft union formation under low night temperatures. These results provide information to develop strategies for improving grafting techniques, as well as scientific instruction for the sensible and efficient management of grafted melon seedlings.

## 2. Results

### 2.1. Anatomical Observation during Graft Union Formation

**Figure 1:**
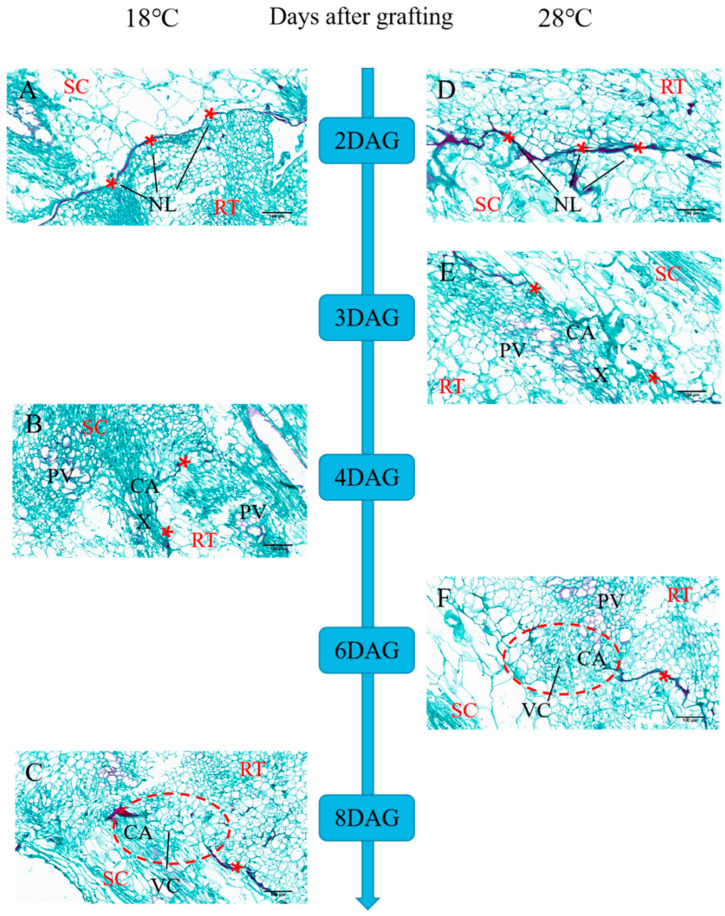
Histological changes of the graft junction using paraffin sectioning and microscopy methods during graft union development under 28 °C and 18 °C night temperatures. (**A**,**D**) Isolation layer stages; (**B**,**E**) the callus phase; (**C**,**F**) the vascular bundle reconnection phase; SC, scion; RT, rootstock; DAG, days after grafting; NL, necrotic layer; VC, vascular bundle reconnection; CA, callus; X, nascent xylem element; PV, original vascular bundle bridge; *, the graft interface; bars, 100 µm.

Graft healing formation was divided into the following three recognizable developmental stages: the isolated layer (IL) stage, the callus (CA) stage, and the vascular bundles (VBs) stage (Figure 1A,D). To investigate the effect of night temperature treatment on graft healing, we conducted a paraffin section test. At 2 DAG, a thin and deep-staining isolation layer was observed. The results show that low temperatures did not affect the formation of an isolated layer. With graft junction development, the isolation layer gradually disappeared. Callus tissue (CA) provides a pathway for communication between the scion and stock. The graft junction under a night temperature of 28 °C formed callus tissue at 3 DAG (Figure 1E), whereas callus formation under a night temperature of 18 °C was observed at 4 DAG (Figure 1B). Vascular connection between the grafted partners was a mark of grafting success. At 6 DAG (Figure 1F), the graft junction under a night temperature of 28 °C formed vascular bundles (VBs), and new vascular bundle formation under a night temperature of 18 °C occurred at 8 DAG (Figure 1C). The results suggest that the healing process of oriental melon scion grafted onto pumpkin rootstock was enhanced under a 28 °C night temperature compared to an 18 °C night temperature.

### 2.2. Reconnection of Vascular Bundles

**Figure 2:**
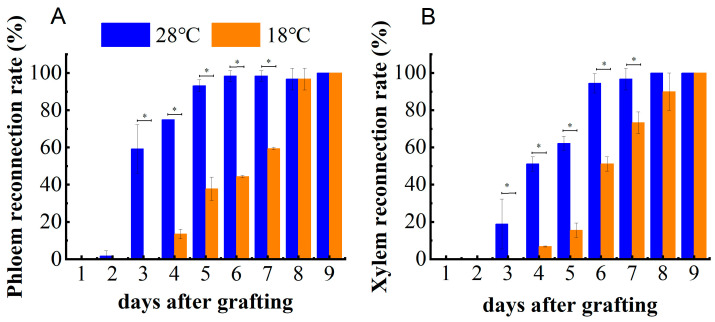
Phloem reconnection rate (**A**) and xylem reconnection rate (**B**) after grafting. Data for each time point were collected from the three treatments with 20 seedlings per treatment and are presented as the mean ± SE. A temperature of 28 °C means that the day and night temperatures were 28 °C/28 °C. A temperature of 18 °C means that the day and night temperatures were 28 °C/18 °C. Asterisks indicate significant differences using a *t*-test (*p* ≤ 0.05).

The Esculin assay was used to monitor phloem connectivity. We applied Esculin solution to the cotyledons and examined the fluorescent signals on a daily basis. Upon comparing the epicotyl of the scion 1 cm above the graft junction with the hypocotyl of the rootstock 1 cm below the graft junction, few grafted individuals exhibited a fluorescence signal in the hypocotyl of the rootstock after the application of Esculin to the cotyledons at 2 DAG with a 28 °C night temperature treatment and 4 DAG with an 18 °C night temperature treatment, respectively. Nearly 90% of the individuals showed a fluorescence signal at 5 DAG under a night temperature of 28 °C and at 8 DAG under a night temperature of 18 °C (Figure 2A).

Next, we soaked the rootstock in 0.1% (*w*/*v*) acid fuchsin solution and monitored the dye in the epicotyl of the scion 1 cm above the graft junction to assay xylem reconnection. Approximately 90% of scions exhibited acid fuchsin dye at 6 DAG under a night temperature of 28 °C and at 8 DAG under a night temperature of 18 °C (Figure 2B). Taken together, the results show that phloem reconnection between the scion and rootstock under a night temperature of 28 °C was established two days earlier than under a night temperature of 18 °C, and xylem reconnection between the scion and rootstock under a night temperature of 28 °C was established one day earlier than under a night temperature of 18 °C.

### 2.3. Variations of Sugars

**Figure 3:**
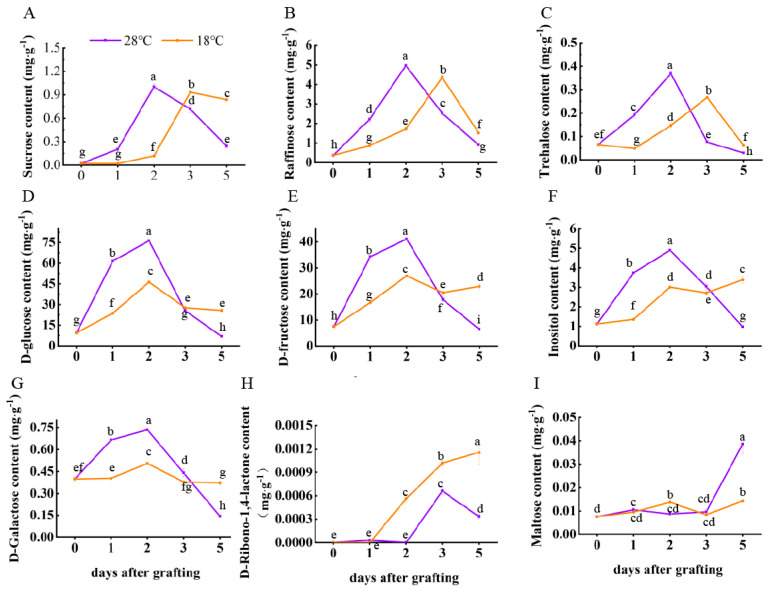
Nine significantly enriched sugar levels. (**A**) Sucrose content; (**B**) raffinose content; (**C**) trehalose content; (**D**) D–glucose content; (**E**) D–fructose content; (**F**) inositol content; (**G**) D–galactose content; (**H**) D–ribono–1, 4–lactone content; and (**I**) maltose content. The different small letters indicate significant differences at the *p* < 0.05 level.

To better understand how elevated temperatures promoted graft formation, we analyzed the content of sugars using an ultra-performance liquid chromatography–electrospray tandem mass spectrometry detection platform. The levels of 29 sugars in metabolic profiles were analyzed throughout the graft union formation process. We compared the metabolic profiles of 28 °C and 18 °C at four stages to identify the DEMs during graft union formation. The DEMs were filtered according to an expression level |log_2_ (fold-change)| > 1 and a *p*-value < 0.05 in each pairwise comparison. Nine sugars, including sucrose, trehalose, maltose, D–ribono–1, 4–lactone, inositol, D–glucose, D–galactose, D–fructose, D–galactose, and raffinose, were significantly enriched in graft union (Figure 3).

The content of sucrose, trehalose, raffinose, D–glucose, D–fructose, D–galactose, and inositol showed an initial increase but then decreased. The content of sucrose, trehalose, and raffinose reached a maximum on the second day after grafting, whereas under a night temperature of 18 °C, they reached a maximum on the third day after grafting (Figure 3A–C). Treatment with a 28 °C night temperature increased the content of sucrose, trehalose, and raffinose significantly at 1 DAG and 2 DAG, but then the content of these sugars decreased significantly at 3 DAG and 5 DAG, compared with 18 °C night temperature treatment. On the second day after grafting, the content of D–glucose, D–fructose, D–galactose, and inositol in two treatments were highest (Figure 3D–G). Furthermore, the content of D–glucose, D–fructose, D–galactose, and inositol under a night temperature of 28 °C was significantly increased, with levels 64.76%, 52.50%, 62.97%, and 26.33% higher than those under a night temperature of 18 °C, respectively.

Grafting induced D–ribono–1, 4–lactone synthesis-related metabolism, leading to an elevated content of D–ribono–1, 4–lactone. However, D–ribono–1, 4–lactone levels under a night temperature of 18 °C were significantly higher than those under a night temperature of 28 °C (Figure 3H). The content of maltose under a night temperature of 28 °C was stable in the early stages (1–3 days after grafting) and significantly increased 5 days after grafting (Figure 3I).

### 2.4. Exogenous Sucrose, Glucose, or Fructose Treatment under 18 °C Night Temperature

**Figure 4:**
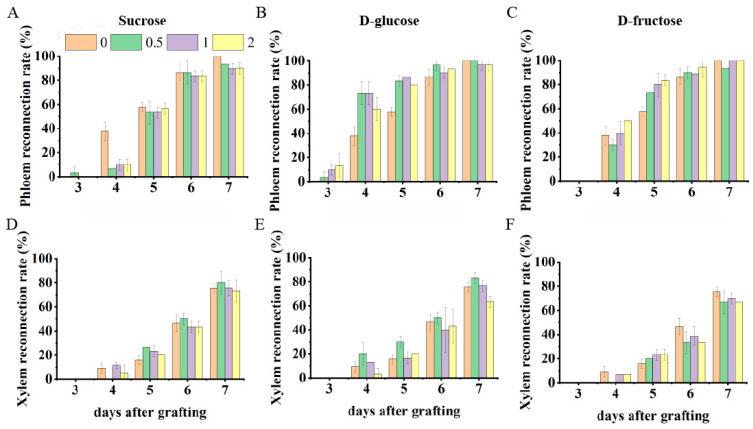
The phloem and xylem reconnection rate of grafted seedlings treated with exogenous sucrose, glucose, and fructose. (**A**,**D**) Exogenous sucrose treatment; (**B**,**E**) exogenous glucose treatment; and (**C**,**F**) exogenous fructose treatment.

To investigate the effects of the level and type of sugars on graft union formation, we sprayed a range of concentrations of exogenous sucrose, glucose, or fructose solution (0, 0.5, 1, and 2%) on the seedling after grafting. The results show that exogenous sucrose application could not promote vascular reconnection. Phloem reconnection was not significantly different under exogenous sucrose treatments (Figure 4A), and the xylem reconnection rate decreased by 466.67%, 277.78%, and 277.78% at 4 DAG under 0.5%, 1%, and 2% exogenous sucrose treatments compared with CK (Figure 4D). During the entire healing period after grafting, the xylem reconnection rate was not significantly different under exogenous sucrose treatments.

Exogenous glucose application promoted vascular reconnection (Figure 4B). The phloem reconnection rate increased by 94.12%, 94.12%, and 58.82% at 4 DAG under 0.5%, 1%, and 2% exogenous glucose treatments compared with CK. The xylem reconnection rate increased by 117.24% at 4 DAG under 0.5% exogenous glucose treatment compared with CK (Figure 4E). However, xylem reconnection was not significantly different under 1% and 2% exogenous glucose treatments.

Exogenous fructose application promoted phloem reconnection. The phloem reconnection rate increased by 26.92%, 38.46%, and 44.23% at 5 DAG under 0.5%, 1%, and 2% exogenous fructose treatments compared with CK (Figure 4C). The xylem reconnection rate was not significantly different under exogenous sucrose treatments (Figure 4F).

## 3. Discussion

Temperature is the key factor for graft union formation. Several reports have shown that the optimal temperature range for healing grafted melons is 28–30 °C [17,18]. Previous studies have shown that when the night healing temperature decreases to 12 °C, there is a wider gully between the rootstock and scion cut surface and the differentiation of vascular tissue is delayed, leading to unsuccessful graft union formation [11]. Furthermore, the effects of different night temperatures on phloem and xylem reconnection were tested. In preliminary tests using 18 °C, 23 °C, and 28 °C, the differences between the phloem and xylem reconnection rates between 18 °C and 23 °C were not significant, but they were significant between 18 °C and 28 °C. Therefore, in this study, 28 °C was set as the high temperature and 18 °C was set as the low temperature to explore the effects of night temperature on the anatomy and sugar contents during the graft healing of melon–pumpkin heterografts.

Sucrose may improve callus formation and the connectivity of vascular bundles at the graft interface. Melnyk et al. [19] found that a level of 0.5% exogenous sucrose enhances the graft survival rate of Arabidopsis. Dabirian and Miles [20] found that drench applications of 1% and 2% sucrose solution to rootstock seedlings before grafting increases the carbohydrate level in the hypocotyl and increases grafting success when both cotyledons are removed from the rootstock before grafting. Miao et al. [14] found that phloem reconnection of cucumber/pumpkin grafts was not significantly different under exogenous glucose treatments, and xylem reconnection was achieved 1 day earlier after grafting with 0.5% exogenous glucose. Differential sugar responses at the graft junction might be important for vascular reconnection. Therefore, to test the effect of different sugars on graft healing, the grafted melon/pumpkin was sprayed with different concentrations of glucose (0.5, 1, and 2%) (*w*/*v*), fructose (0.5, 1, and 2%) (*w*/*v*), and sucrose (0.5, 1, and 2%) (*w*/*v*) when grafting.

### 3.1. Elevated Temperature Improves the Process of Graft Union Formation

Graft healing is a complex process that is affected by environmental factors, with temperature being one of the most important influencing factors. Previous work has described the stages of graft union formation, including the production of a necrotic layer, the proliferation of callus cells at the graft interface, and vascular redifferentiation across the graft interface [7,19,21]. The connection of the graft union between rootstock and scion is influenced by plant growth conditions, age, species, and so on. It takes an average of 5 to 14 d for the graft union of herbaceous plants to develop vascular connectivity between rootstock and scion [22,23].

Vascular tissue reconnection is considered an important indicator of grafting success. A robust vascular connection between the scion and the rootstock determines the physiological functionality of the plant, influencing vital processes such as water and nutrient uptake and translocation, which in turn regulate nutrition, organ growth, photosynthesis, and transpiration [24]. Oriental melon grafted onto squash showed union bridges between the rootstock and scion 9 days after grafting under 26 ± 3 °C temperature [25], and watermelon grafted onto squash showed that vascular connections appeared 5 days after grafting under a night temperature of 18 °C [11]. In this study, functional phloem connection occurred two days earlier than xylem connection when treated with a night temperature of 28 °C. This is consistent with what has been reported for grafted cucumber [14]. However, functional phloem and xylem connection occurred on the same day under a night temperature of 18 °C. These results imply that elevated temperatures improve the process of graft union formation.

### 3.2. The Levels of Nine Sugars Affect Graft Union Formation

A complex network of physiological responses takes place during the formation of the graft union and collectively determine the timeline and outcome of the procedure. Grafting causes mechanical damage to the plant, which sets off responses to counteract the damage, including the inhibition of cell elongation, stomatal closure, and reductions in transpiration, stomatal conductance, and CO_2_ assimilation. Furthermore, there is an accumulation of reactive oxygen species (ROS) [26,27,28]. These ROS have the potential to cause oxidative damage. Accumulated soluble sugars can protect themselves against potential oxidative damage, including cellular membrane collapse and the breakdown of cellular compartments [29].

Achieving homeostasis allows the plant to rebuild tissues and forge a union between the two components of the graft. Tissue adhesion begins with the accumulation of polysaccharides, such as cellulose and pectin [30,31]. Carbon skeletons are required in the damaged area for the synthesis of new molecules [32]. Simultaneously, callus-like masses of undifferentiated cells (known as callus tissue) proliferate adjacent to the existing vascular tissue [33]. Furthermore, cell proliferation is an energy-consuming process. Sugars, as an energy source, provide ATP through glycolysis pathways and cellular respiration, and eventually activate cell metabolism. Sugars are actively involved in cell division and cell expansion [14,34]. Additionally, sugars, as signaling molecules, may regulate gene expression and a variety of metabolic processes through hexokinase-dependent and independent pathways. Pu et al. [35] found that the starch content in the rootstock cotyledons of cucumber grafted onto pumpkin seedlings decreased during 0–3 days after grafting. Amri et al. [36] discovered a significant increase in soluble sugar and starch content at the rootstock and scion union in peach/plum grafting. In our data sets, nine sugars were significantly increased. However, the varying trends observed in the levels of the nine sugars may have resulted in the abnormal formation of vascular tissue.

The accumulation of sugars in sink tissues depends on the actions of several genes related to biosynthesis, metabolism, and transportation. In *Cucurbitaceae*, raffinose and stachyose are the predominant carbohydrates transported from leaves to sink tissues [37]. In sink tissues, raffinose family oligosaccharides (RFOs) are unloaded from the phloem, and alkaline α-galactosidases hydrolyze the RFOs to sucrose and galactose. When a plant suffers stress, sucrose will be used for long-distance transport [38]. In this study, the change in sucrose content was similar to that of raffinose, but different from that of D–galactose. So, sucrose and raffinose were the main carbohydrates transported from leaves to the graft union. The exogenous application of sucrose did not promote vascular reconnection.

Sucrose can be converted to its hexose monomers (glucose and fructose) by invertases or converted to fructose and uridine diphosphate-glucose (UDPG) by sucrose synthase [39]. The metabolic processes of sucrose are mainly regulated by sucrose–phosphate synthase (SPS), sucrose–phosphate phosphatase (SPP), and sucrose synthase (SUS). The content of D–glucose, D–fructose, D–galactose, and inositol in two treatments were highest 2 days after grafting. However, the contents of D–glucose, D–fructose, D–galactose, and inositol under a night temperature of 28 °C were significantly higher than those under a night temperature of 18 °C. Exogenous glucose application promoted vascular reconnection, and exogenous fructose application promoted phloem reconnection. Therefore, high night temperatures accelerate graft union formation by promoting the accumulation of glucose and fructose. However, further study is required to understand how glucose and fructose regulate graft union formation.

## 4. Materials and Methods

### 4.1. Plant Materials

Scion: melon (*Cucumis melo* L.), Meinong Cultivar, was obtained from Hubei Xueyin Agricultural Technology Co., Ltd., Jingzhou, China.

Rootstock: pumpkin (*Cucurbita moschata* Dutch.), Zhenzhuang Cultivar, was obtained from Jingyan Yinong Seed Sci-Tech Co., Ltd., Shouguang, China.

All the experiments were carried out in an artificial chamber in 2022 at the Wuhan Agricultural Academy, Central China (30°27′ N, 114°20′ E, and at an altitude of 22 m above sea level). The scion and rootstock seeds were sown into 98- and 72-cell trays, respectively, with one seed in one cell filled with mixed seedling substrate (peat moss and pearlite at a volume ratio of 3:1). The melon seeds were sown 5 days before the pumpkin seeds and were placed in a germination chamber at 30 °C for 2 days. After germination, the melon and pumpkin seedlings were transferred to the artificial chamber. The plants were cultivated with a day/night (12 h/12 h) cycle at 28 °C/18 °C and 60–80% relative humidity. The plants were fertilized with a water-soluble fertilizer (Product Model: 20-10-20 + TE, 1000 times liquid, Shanghai Yongtong chemical Co., Ltd., Shanghai, China).

### 4.2. Grafting and Temperature Treatment

The melon scions were grafted onto pumpkin rootstock using the root-pruning splice grafting technique at the first-true-leaf development stage [2]. After grafting, the grafted melon seedlings were divided into two groups and were placed in a hydroponic box containing 1/2 Hoagland’s nutrient solution (12 cm × 12 cm) for continuous temperature treatment. One group was placed in an artificial chamber with a temperature of 28 °C day/28 °C night (28 °C), and another group was placed in an artificial chamber with a temperature of 28 °C day/18 °C night (18 °C). The other conditions included a photoperiod of 12 h day/12 h night, a light intensity of 50 μM·m^−2^·s^−1^, and a constant humidity of 95–100%. Three separate biological experiments were conducted under the same conditions to replicate the results.

### 4.3. Exogenous Glucose, Fructose, and Sucrose Treatments

To test the effects of different sugars on graft healing, the grafted melon/pumpkin was sprayed with different concentrations of glucose (0.5, 1, and 2%) (*w*/*v*), fructose (0.5, 1, and 2%) (*w*/*v*), and sucrose (0.5, 1, and 2%) (*w*/*v*) when grafting. Distilled water was sprayed as the control. Three hundred plants were used for each treatment. Phloem and xylem connectivity was observed 3, 4, 5, 6, and 7 d after grafting. Twenty plant replicates were performed for every treatment. Three separate biological experiments were conducted under the same conditions to replicate the results.

### 4.4. Paraffin Sectioning and Microscopy

A sample of 0.3 cm stems above and below the graft junction was collected at 1–9 DAG. The collected samples were fixed, softened, dehydrated, infiltrated, and embedded in paraffin, as described by Ribeiro et al. [40]. The samples were sectioned to 10 μm vertically using a rotary microtome (RM2016; Leica; Shanghai, China), dewaxed, rehydrated, cleaned, stained with Fast Green, counterstained with safranin, and then fixed with neutral balata. The sections were examined using a light microscope (NIKON DS-U3; Nikon Corp., Tokyo, Japan), and representative sections were photographed.

### 4.5. Assays of Phloem and Xylem Connectivity

Phloem and xylem connectivity were measured by Esculin and acid fuchsin movement across the graft union, respectively [41]. To assay phloem connections, 0.4 g of Esculin was dissolved in 20 mL of 60% Acetonitrile. The cotyledon center of the scion was gently scraped with a sharp single-edge razor to create a small opening for the Esculin to enter. Next, a 30 μL Esculin sample was added to each cotyledon, and fluorescence in the rootstock hypocotyls was measured after 2 h of incubation. For xylem connection, the plant roots were incubated in a solution of 5 mg/mL of acid fuchsin, and the melon hypocotyls were examined after 2 h.

The phloem and xylem reconnection rates were investigated using the following formulae:

Phloem reconnection rate (%) = (phloem reconnected grafts/total test grafts) × 100%

Xylem reconnection rate (%) = (xylem reconnected grafts/total test grafts) × 100%

### 4.6. Determination of Sugar Contents

The samples were harvested from the grafting union at 0, 1, 2, 3, and 5 DAG. The sugars were extracted and assayed according to previous reports [42]. All the metabolites were detected using MetWare (http://www.metware.cn/, accessed on 25 September 2022) on the AB Sciex QTRAP 6500 LC-MS/MS platform. After the sample was thawed and smashed, 0.05 g of the sample was mixed with 500 µL of 70% methanol/water. The sample was vortexed for 3 min under 2500 r/min and centrifuged at 12,000 r/min for 10 min at 4 °C. A total of 300 μL of supernatant was transferred into a new centrifuge tube and placed in a −20 °C refrigerator for 30 min. Then, the supernatant was centrifuged again at 12,000 r/min for 10 min at 4 °C. After centrifugation, 200 μL of supernatant was transferred through a Protein Precipitation Plate for further LC-MS analysis. The sample extracts were analyzed using an LC-ESI-MS/MS system (Waters ACQUITY H-Class, https://www.waters.com/nextgen/us/en.html, accessed on 18 December 2022; MS, QTRAP^®^ 6500+ System, https://sciex.com/, accessed on 20 December 2022). Three separate biological experiments were conducted under the same conditions to replicate the results.

### 4.7. Statistical Analysis

The data are presented as the mean ± standard deviation of three replicate samples. Significantly regulated metabolites between the groups were determined using VIP and absolute Log_2_FC (fold change). The statistical evaluations were performed via one-way analysis of variance, followed by individual comparisons with *t*-tests (*p* < 0.05) using SAS 9.0.3 software.

## 5. Conclusions

The graft healing process takes longer and involves higher costs in early spring, when plants often suffer from low temperature exposure. Temperature is undoubtedly the key factor affecting graft union formation. Elevated temperatures improve the process of graft union formation by increasing the content of sucrose, trehalose, maltose, D–ribono–1, 4–lactone, inositol, D–glucose, D–galactose, D–fructose, D–galactose, and raffinose. Moreover, exogenous glucose and fructose application promotes vascular reconnection. However, exogenous sucrose application does not promote vascular reconnection. These results provide information to develop strategies for improving grafting efficiency under low temperatures.

## Data Availability

The data presented in this study are available upon request from the first author at lianghuanconf@126.com.
